# Development and Validation of a Novel PPAR Signaling Pathway-Related Predictive Model to Predict Prognosis in Breast Cancer

**DOI:** 10.1155/2022/9412119

**Published:** 2022-06-02

**Authors:** Yingkun Xu, Dan Shu, Meiying Shen, Qiulin Wu, Yang Peng, Li Liu, Zhenrong Tang, Shun Gao, Yuan Wang, Shengchun Liu

**Affiliations:** Department of Endocrine and Breast Surgery, The First Affiliated Hospital of Chongqing Medical University, Chongqing 400042, China

## Abstract

This study is aimed at exploring the potential mechanism of the PPAR signaling pathway in breast cancer (BRCA) and constructing a novel prognostic-related risk model. We used various bioinformatics methods and databases to complete our exploration in this research. Based on TCGA database, we use multiple extension packages based on the R language for data conversion, processing, and statistics. We use LASSO regression analysis to establish a prognostic-related risk model in BRCA. And we combined the data of multiple online websites, including GEPIA, ImmuCellAI, TIMER, GDSC, and the Human Protein Atlas database to conduct a more in-depth exploration of the risk model. Based on the mRNA data in TCGA database, we conducted a preliminary screening of genes related to the PPAR signaling pathway through univariate Cox analysis, then used LASSO regression analysis to conduct a second screening, and successfully established a risk model consisting of ten genes in BRCA. The results of ROC curve analysis show that the risk model has good prediction accuracy. We can successfully divide breast cancer patients into high- and low-risk groups with significant prognostic differences (*P* = 1.92*e* − 05) based on this risk model. Combined with the clinical data in TCGA database, there is a correlation between the risk model and the patient's N, T, gender, and fustat. The results of multivariate Cox regression show that the risk score of this risk model can be used as an independent risk factor for BRCA patients. In particular, we draw a nomogram that can predict the 5-, 7-, and 10-year survival rates of BRCA patients. Subsequently, we conducted a series of pancancer analyses of CNV, SNV, OS, methylation, and immune infiltration for this risk model gene and used GDSC data to investigate drug sensitivity. Finally, to gain insight into the predictive value and protein expression of these risk model genes in breast cancer, we used GEO and HPA databases for validation. This study provides valuable clues for future research on the PPAR signaling pathway in BRCA.

## 1. Introduction

According to the latest global cancer burden data, there are 2.26 million new breast cancer cases (BRCA), surpassing lung cancer and becoming the world's largest cancer type [1]. The mortality rate of BRCA is also very high. More than 600,000 deaths from BRCA seriously endanger human beings' physical and mental health [2]. As a heterogeneous disease, the pathogenesis of BRCA is complex, and its occurrence and development are the results of the joint action of multiple genes, and the specific etiology is still unclear [3]. Some scholars believe that its occurrence may be closely related to high-risk factors such as long menstrual cycles and high primiparous age [4, 5]. Similar to other solid malignant tumors, tumor invasion, metastasis, and spread are undoubtedly the most common cause of death in BRCA patients [6–8]. Common sites of BRCA metastases include the liver, brain, bone, or lung [9–12]. Studies have shown that BRCA patients with distant metastasis have a poor prognosis, high recurrence rate, and short survival time [13–15]. With the application of surgery, chemotherapy, radiotherapy, endocrine therapy, immunotherapy, and a variety of combined treatment strategies, the survival time of BRCA patients has been significantly prolonged [16–18]. However, there are still many BRCA patients who eventually develop recurrence, metastasis, and drug resistance. Therefore, the search for effective biomarkers and new therapeutic targets will play a vital role in the future treatment and prognosis of BRCA.

Since it was first discovered in the 1990s, the peroxisome proliferator-activated receptor (PPAR) signaling pathway has received wide attention from researchers [19]. As researchers continue to explore and research, it has been determined that the PPAR signaling pathway plays a crucial role in cell differentiation, inflammation, glucose and lipid metabolism, immune regulation, and tumorigenesis [20, 21]. PPARs play an essential mediating role in the PPAR signaling pathway. PPARs are nuclear hormone receptors activated by fatty acids and their derivatives and belong to the ligand-activated receptors in the nuclear hormone receptor family [22]. PPARs are composed of three subtypes: PPAR*α*, PPAR*β*, and PPAR*γ*. PPARs, like other nuclear receptor superfamilies, are essentially a type of ligand-dependent transcriptional regulators. Many studies have shown that the abnormal regulation of the PPAR signaling pathway is usually accompanied by the occurrence and development of various cancers, such as bladder cancer, astroglioma, renal clear cell carcinoma, hepatocellular carcinoma, and colorectal cancer [23–28]. In BRCA, studies have identified the PPAR signaling pathway as a highly relevant biological process [29–31]. Still, there is no research to screen the prognostic markers of BRCA from the PPAR signaling pathway.

**Figure 1:**
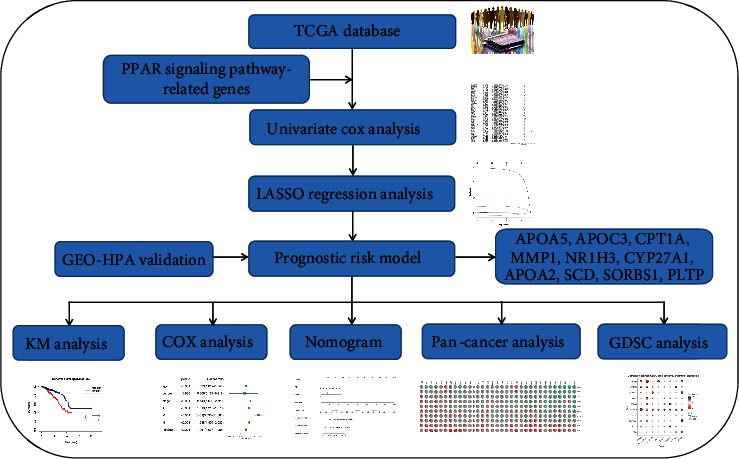
The flow chart of this research.

Based on the background of the above research, this research is dedicated to exploring the genes related to the PPAR signaling pathway to construct a novel prognostic risk model in BRCA. Based on the gene expression and clinical information data of 1,098 BRCA patients contained in TCGA database, we used univariate Cox regression analysis and LASSO regression analysis to select ten genes from 69 PPAR signaling pathway-related genes (APOA2, APOA5, APOC3, CPT1A, CYP27A1, MMP1, NR1H3, PLTP, SCD, and SORBS1) to build a novel risk model. We successfully divided BRCA patients into high- and low-risk groups with significant prognostic differences based on this risk model. Next, we use ROC curve to evaluate the predictive accuracy of the risk model. At the same time, we analyze the correlation between this risk model and clinicopathological characteristics and draw the corresponding heat map. In addition, to improve the possibility of clinical application of the risk model, we have established a corresponding nomogram. Subsequently, we conducted a series of pancancer analyses to explore this risk model gene's potential clinical application prospects. Finally, we use the protein expression of risk model genes in BRCA and normal breast tissues to verify our previous findings. In particular, to make it easier for readers to understand this research, we draw a flowchart of this research (Figure 1). In future clinical diagnosis and treatment, we believe that the prognostic-related risk model constructed by this research may help doctors more accurately identify patients with poor prognoses and provide more targeted treatment and examination. This research also provides valuable data support for future research on the PPAR signaling pathway in BRCA.

## 2. Materials and Methods

### 2.1. Data Acquisition

TCGA, as the most prominent cancer gene information database, covers 33 cancer types, more than 30,000 tumor samples, and multiple omics data (including gene expression data, miRNA expression data, gene mutation, and DNA methylation) [32]. In this study, we downloaded and compiled BRCA patient transcriptome expression profile data and patient clinical data from TCGA database in Chongqing in September 2021 (https://portal.gdc.cancer.gov). In addition, we inquired and obtained a list of genes related to the PPAR signaling pathway through GSEA website (https://www.gsea-msigdb.org/gsea/index.jsp) [33, 34]. Its standard name is KEGG PPAR SIGNALING PATHWAY, and its systematic name is M13088.

### 2.2. Data Processing and Analysis

First, we downloaded BRCA's RNA-Seq transcriptome data and clinical information through TCGA database and used Perl and R language to process the data and draw the figure. Among them, we use the mRNA expression data of TCGA to draw a heat map showing the expression of genes related to the PPAR signaling pathway in BRCA, “pheatmap” package is used to draw the heat map, and “limma” package is used to analyze mRNA expression differences. Subsequently, we performed hazard ratio analysis of these molecules in BRCA to show the relationship between these molecules and the progression of BRCA. After that, we use “glmnet” and “survival” packages to draw the LASSO regression curve and the corresponding survival curve. To verify the prediction accuracy of this model, we used “survivalROC” extension package to draw ROC curves for 5, 7, and 10 years. And then, we analyzed the correlation between the risk model and the pathological characteristics of BRCA patients. In particular, we combine the clinical data of BRCA patients with risk models through “survival” and “forestplot” packages to perform univariate and multivariate Cox analysis. Finally, to be more convenient for future clinical applications, we use “rms” software package to draw the corresponding nomogram.

### 2.3. GEPIA and GSCA Website

GEPIA website is a visual website developed and researched by Peking University that integrates database information (http://gepia2.cancer-pku.cn/#index) [35]. We used the online tools of the website to analyze the OS of the risk model genes in pancancer and presented the corresponding results. In addition, GSCA website is an integrated database for genomic and immunogenomic gene set cancer analysis. Cancer researchers often use it for dynamic analysis and visualization of cancer genomes and to determine the correlation with multiple anticancer drugs (http://bioinfo.life.hust.edu.cn/GSCA/#/). In this study, we used the online tools of the website to analyze the CNV and SNV of the risk model genes in pancancer and presented the corresponding results.

### 2.4. ImmuCellAI Database

Immune Cell Abundance Identifier (ImmuCellAI) database, as a network platform for comprehensive analysis of immune cell abundance, estimates the infiltration abundance of 24 immune cells based on gene expression data sets, including RNA-Seq and microarray data (http://bioinfo.life.hust.edu.cn/ImmuCellAI#!/). At the same time, it can predict the patient's response to immune checkpoint inhibitor therapy [36, 37]. This study obtained 24 immune cell infiltration data through the ImmuCellAI website. In addition, gene set variation analysis (GSVA) score represents the comprehensive level of gene set expression and is positively correlated with gene set expression. The “GSVA” package calculates the GSVA score in this study in the R language. The correlation between immune cell infiltration and the GSVA score of the risk model gene set was expressed by the correlation coefficient, which was evaluated by Spearman correlation analysis. FDR adjusted the *P* value.

### 2.5. TIMER Database

Tumor Immune Estimation Resource (TIMER) database is an interactive web tool that can comprehensively and flexibly analyze and visualize tumor-infiltrating immune cells and infer the abundance of multiple tumor-infiltrating immune cells from the gene expression profiles of samples of different cancer types in TCGA, such as B cells, CD4+ T cells, CD8+ T cells, neutrophils, macrophages, and dendritic cells (https://cistrome.shinyapps.io/timer/) [38, 39]. In this study, we used the mRNA expression data of the risk model gene in TCGA, combined the immune cell infiltration data in the TIMER database, explored the concern between gene expression and immunity in BRCA, and used the R language “pheatmap” package to draw the corresponding heat map.

### 2.6. GDSC Database

GDSC database collects the sensitivity and response of tumor cells to drugs (https://www.cancerrxgene.org/). The data in the GDSC database comes from 75,000 experiments, describing the reaction of about 200 anticancer agents in more than 1,000 tumor cells. We collected IC50 of 265 small molecules in 860 cell lines and its corresponding mRNA gene expression from GDSC. The mRNA expression data and drug sensitivity data were merged. Pearson correlation analysis was performed to correlate gene mRNA expression and drug IC50. FDR adjusted the *P* value.

### 2.7. The Human Protein Atlas Database

The Human Protein Atlas database provides information on the tissue and cell distribution of all 24,000 human proteins (https://www.proteinatlas.org/) [40–42]. The database uses immunohistochemical technology to examine the distribution and expression of each protein in a variety of human normal tissues, tumor tissues, and cell lines. The results are read and indexed by professionals. We used immunohistochemical data to explore the expression of these risk model genes in BRCA tissues and normal breast tissues.

## 3. Results

### 3.1. The Expression of PPAR Signaling Pathway-Related Genes in BRCA and Univariate Cox Analysis

**Figure 2:**
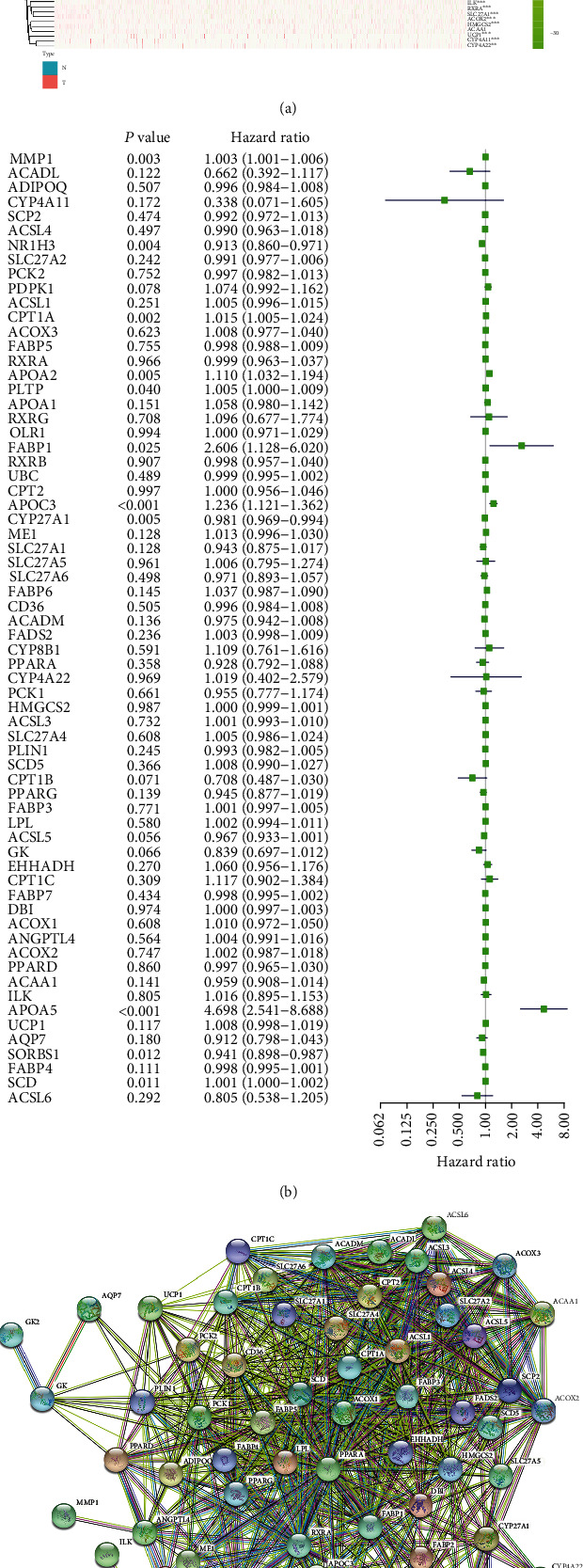
The expression of genes related to the PPAR signaling pathway in BRCA and univariate Cox regression analysis. (a) The heat map shows the expression of genes related to the PPAR signaling pathway in BRCA. ^∗^*P* < 0.05, ^∗∗^*P* < 0.01, and ^∗∗∗^*P* < 0.001. (b) The forest plot shows the results of univariate Cox analysis of PPAR signaling pathway-related genes in BRCA. (c) PPAR signaling pathway-related molecular interaction network diagram.

To understand the expression of genes related to the PPAR signaling pathway in BRCA, based on the mRNA expression data in TCGA database, we draw the corresponding heat map with the help of “pheatmap” package in the R language. We can see a significant difference in the expression of most genes between BRCA tissue and normal breast tissue (Figure 2(a)). We infer that the abnormality of the PPAR signaling pathway may play an essential role in the occurrence and development of BRCA. Subsequently, the results of univariate Cox analysis showed that APOA5, APOC3, CPT1A, MMP1, APOA2, SCD, FABP1, and PLTP played risk factors in BRCA progression, while NR1H3, CYP27A1, and SORBS1 played protective factors in BRCA progression (Figure 2(b)). So far, we have used univariate Cox analysis to select eleven genes (including APOA5, APOC3, CPT1A, MMP1, APOA2, SCD, FABP1, PLTP, NR1H3, CYP27A1, and SORBS1) that play a crucial role in the occurrence and development of BRCA among the genes related to the PPAR signaling pathway. In addition, to explore the relationship between these PPAR signaling pathway-related molecules, we used the STRING database to draw the interaction network map between these molecules (Figure 2(c)).

### 3.2. Construct a Novel Prognostic-Related Survival Model in BRCA

**Figure 3:**
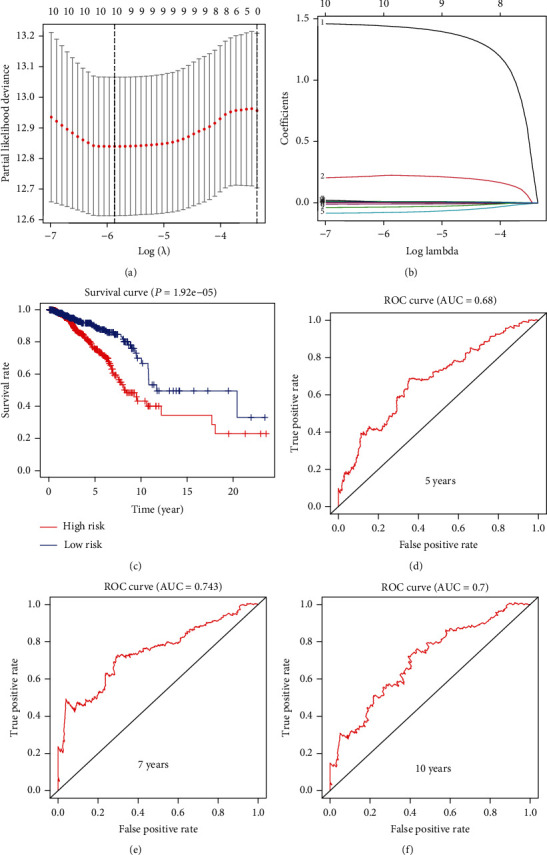
Construct a prognostic-related risk model in BRCA through LASSO regression analysis. (a, b) LASSO coefficient values and vertical dashed lines were calculated at the best log (lambda) value, and coefficients of prognostic-related genes were displayed. (c) Based on the above risk model, the K-M curve in BRCA patients showed that the overall survival rate of the low-risk group was significantly higher than that of the high-risk group (*P* = 1.92*e* − 05). It is worth noting that blue represents the low-risk group, and red represents the high-risk group. (d–f) ROC curves were drawn based on the risk model, and their AUC value represented the 5-, 7-, and 10-year OS of BRCA patients.

To further explore the potential role of PPAR signaling pathway-related genes in BRCA, we used LASSO regression analysis to establish a PPAR signaling pathway-related risk model in BRCA (including APOA5, APOC3, CPT1A, MMP1, NR1H3, CYP27A1, APOA2, SCD, SORBS1, and PLTP) (Figures 3(a) and 3(b)). Subsequently, based on this risk model, we divided BRCA patients into high- and low-risk groups and combined the survival information of the patients to draw a corresponding survival curve (Figure 3(c)). The results of the survival curve showed that the prognosis of patients in the high-risk group was significantly worse than that of the patients in the low-risk group (*P* = 1.92*e* − 05). We have drawn five-year, seven-year, and ten-year ROC curves based on this risk model to test this risk model's prediction accuracy. The five-year, seven-year, and ten-year AUC values are 0.68, 0.743, and 0.7, respectively (Figures 3(d) and 3(f)). The results show that the prognostic-related risk model has good predictive accuracy.

### 3.3. The Relationship between the Risk Model and Clinicopathological Characteristics Draws the Corresponding Nomogram in BRCA

**Figure 4:**
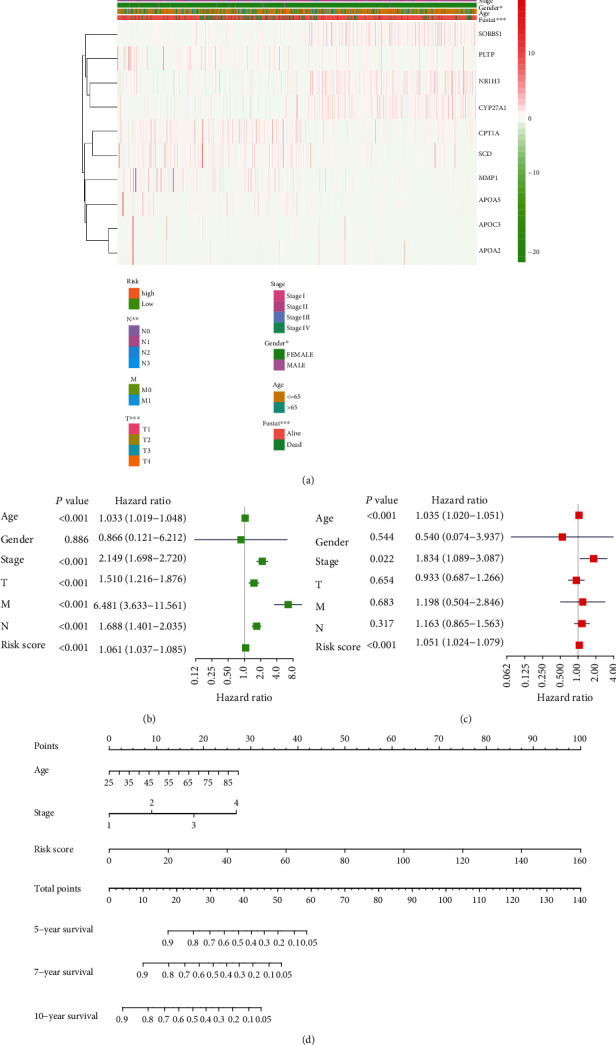
The comprehensive analysis is based on the clinical information of BRCA patients. (a) The heat map shows the correlation between the risk model and the clinicopathological characteristics of BRCA patients. (b) The forest plot shows the results of univariate Cox analysis. (c) The forest plot shows the results of multivariate Cox analysis. (d) Based on this risk model, draw a nomogram that can predict the 5-, 7-, and 10-year OS of BRCA patients.

To better understand the correlation between the risk model and clinical information, we draw a heat map reflecting the correlation between the risk model and the clinicopathological characteristics of BRCA patients. This heat map shows a significant correlation between the risk model and the N, T, gender, and fustat of BRCA patients (Figure 4(a)). Subsequently, the results of univariate Cox analysis showed that age, stage, T, M, N, and risk score played risk factors in BRCA progression (Figure 4(b)). In addition, the results of multivariate Cox analysis showed that age, stage, and risk score played independent risk factors in BRCA progression (Figure 4(c)). In particular, based on the risk model, we draw a nomogram that can predict the overall survival rates of BRCA patients at five, seven, and ten years (Figure 4(d)). We believe this will facilitate the clinical application of this risk model in the future.

### 3.4. Variation and OS of Model Genes in Pancancer

**Figure 5:**
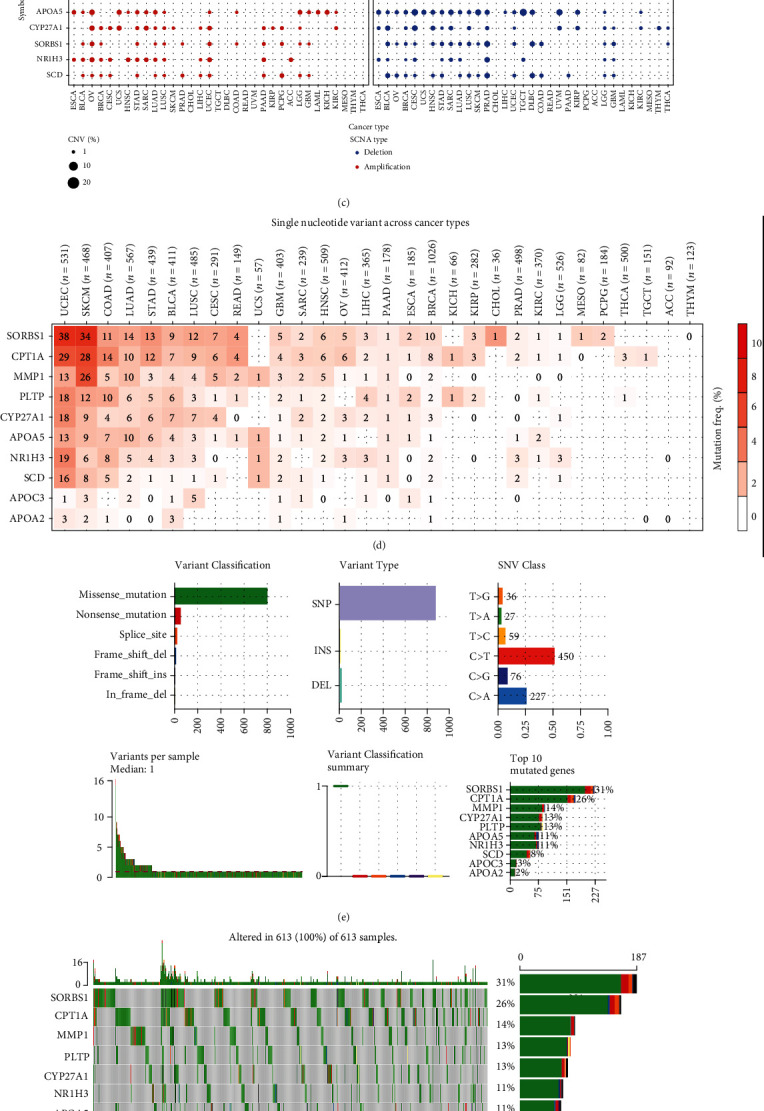
Variation analysis and overall survival analysis of this risk model gene in pancancer. (a–c) CNV of the risk model genes in pancancer. The light red Hete Amp represents heterozygous amplification, the light green Hete Del means heterozygous deletion, the dark red Homo Amp represents homozygous amplification, the dark green Homo Del represents homozygous deletion, and the gray represents no CNV occurrence. (d–f) SNV of the risk model genes in pancancer. The redder the color, the higher the mutation rate. (g) Survival landscape of the risk model genes in pancancer.

To gain a deeper understanding of the potential role of these risk model genes in tumorigenesis, we conducted a series of pancancer analyses. First, we explored the CNV of these risk model genes in 33 different cancers. This heat map shows that these risk model genes have a wide range of CNV in UCS, LUSC, and BRCA, while APOA2, PLTP, and CPT1A have a wide range of CNV in pancancer (Figures 5(a)–5(c)). Subsequently, we investigated the SNV status of these risk model genes in pancancer. SORBS1 and CPT1A genes have high SNV in UCEC and SKCM tumors (Figures 5(d)–5(f)). Finally, we conducted OS analysis for these risk model genes in pancancer and drew the corresponding heat map. It can be seen that CYP27A1 plays a protective factor in ACC, BRCA, KIRC, LIHC, LUAD, and MESO (Figure 5(g)). We believe that these results can provide some clues for future scientific research.

### 3.5. Immune Infiltration, Methylation, and Drug Sensitivity of Model Genes in Pancancer

**Figure 6:**
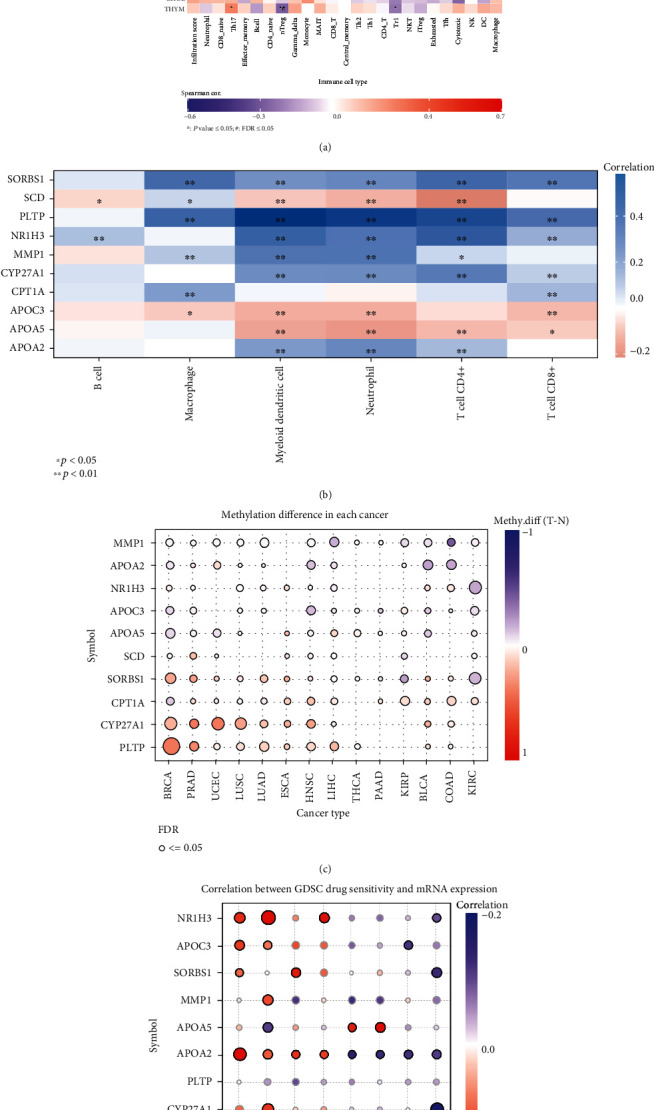
Immune infiltration analysis, methylation analysis, and drug sensitivity analysis of this risk model gene in pancancer. (a) The heat map shows the correlation between the risk model gene and immune cell infiltration in pancancer. It is worth noting that red represents positive correlation and purple represents negative correlation. ^∗^*P* ≤ 0.05 and ^#^FDR ≤ 0.05. (b) The heat map shows the correlation between the risk model gene and immune cell infiltration in BRCA. Blue represents positive correlation, and pink represents negative correlation. ^∗^*P* < 0.05 and ^∗∗^*P* < 0.01. (c) This shows the difference in methylation of these risk model genes in multiple tumor types. (d) This shows the correlation between the risk model genes and the sensitivity of multiple anticancer drugs. Blue bubbles represent negative correlations, and red bubbles represent positive correlations; the deeper of color, the higher the correlation. There is a positive correlation between bubble size and FDR significance. The black outline frame indicates FDR ≤ 0.05.

Since previous studies have shown that the PPAR signaling pathway is closely related to tumor immunity [43, 44], we aimed at this PPAR signaling pathway-related risk model to explore the immune correlation in pancancer. Combined with the immune cell infiltration data in the ImmuCellAI database, we have drawn a heat map reflecting the correlation of this risk model with a variety of immune cell infiltration in pancancer. By reading the heat map, we can find that this risk model has a significant positive correlation with the infiltration score of most tumors. The risk model has a significant positive correlation with macrophage, DC, and NK cell infiltration. On the contrary, it has a significant negative correlation with neutrophil, CD8-naive, and Th17 cell infiltration (Figure 6(a)). To further understand the correlation between these risk model genes and immune cell infiltration in BRCA, we used the data in the TIMER database to draw a corresponding heat map. We can see that SORBS1, PLTP, and NR1H3 are significantly positively correlated with various immune cell infiltration in BRCA. At the same time, SCD, APOC3, and APOA5 are significantly negatively associated with multiple immune cell infiltration in BRCA (Figure 6(b)). In addition, we used TCGA data to explore the differences in methylation of these risk model genes in a variety of tumors. The results show that these risk model genes have extensive methylation differences in BRCA, PRAD, HNSC, and LIHC (Figure 6(c)). Because the mutation of the cancer genome will affect the effect of clinical treatment, the response of different targets to drugs is also very different. Therefore, this data is essential for discovering potential tumor treatment targets. Consequently, we use the data in the GDSC database to explore the sensitivity of these risk model genes to different anticancer drugs. The results show a significant negative correlation between APOA2 and erlotinib sensitivity, while a significant positive correlation exists between APOA5 and lapatinib sensitivity (Figure 6(d)). We believe that our research provides ideas for future drug target development.

### 3.6. Verify the Predictive Value and Protein Expression of Model Genes between BRCA and Normal Breast Tissues

**Figure 7:**
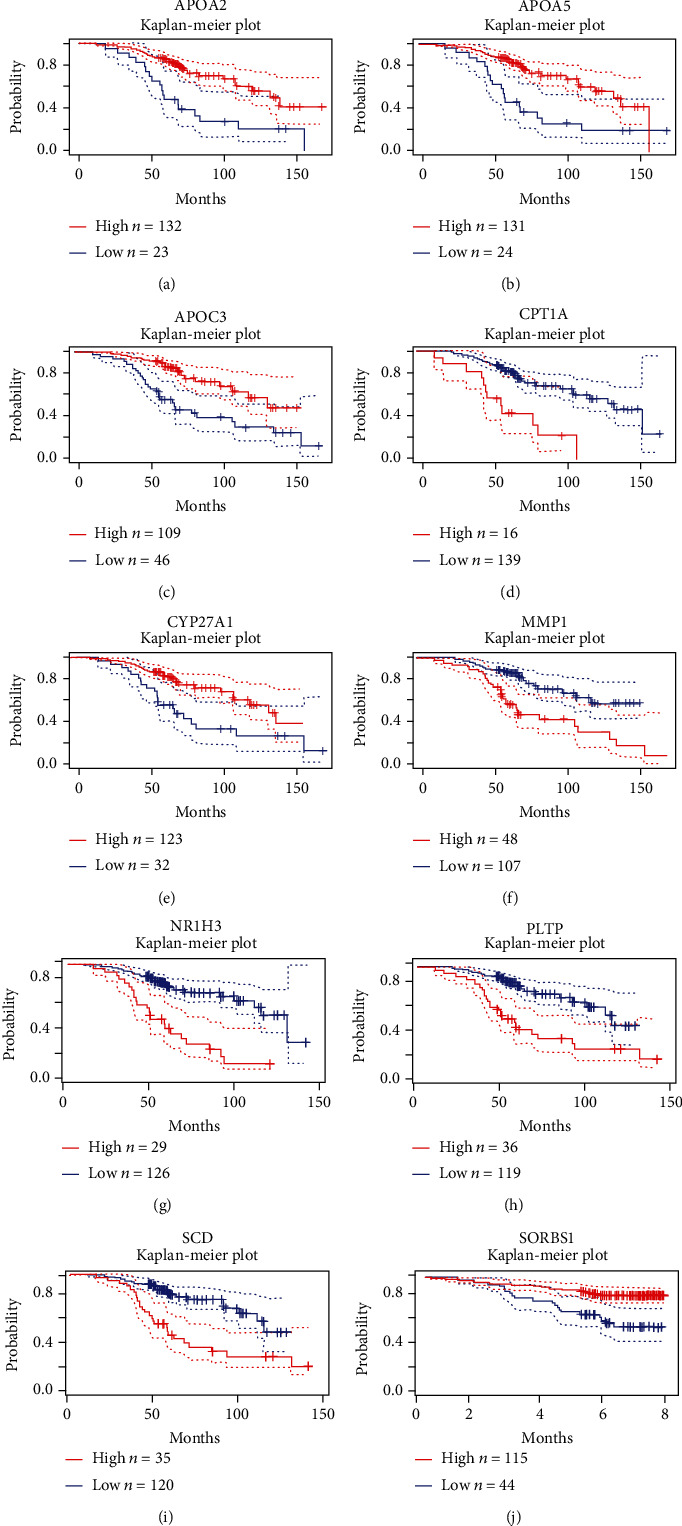
Overall survival curves based on risk model genes in BRCA. (a–j) APOA2, APOA5, APOC3, CPT1A, CYP27A1, MMP1, NR1H3, PLTP, SCD, and SORBS1.

**Figure 8:**
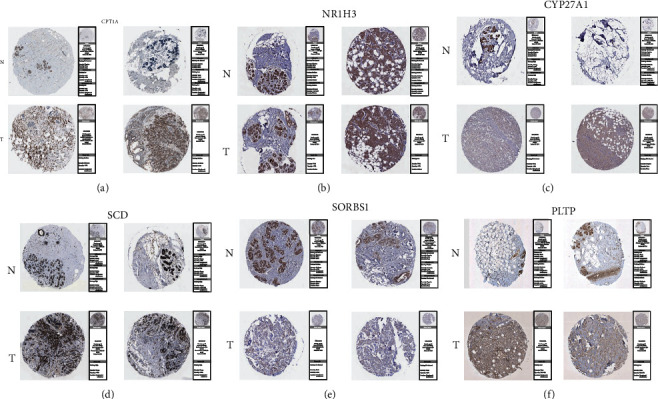
The results of immunohistochemistry. (a–f) The immunohistochemistry results showed the protein expression of CPT1A, NR1H3, CYP27A1, SCD, SORBS1, and PLTP in BRCA tissues and normal breast tissues.

To verify the prognostic value of this risk model gene in BRCA, we used the GEO database (GSE9893 and GSE1456) to draw survival curves in BRCA for APOA2, APOA5, APOC3, CPT1A, CYP27A1, MMP1, NR1H3, PLTP, SCD, and SORBS1 (Figures 7(a)–7(j)). The results showed that BRCA patients with high expression of APOA2, APOA5, APOC3, CYP27A1, and SORBS1 had a higher overall survival rate. In contrast, BRCA patients with high expression of CPT1A, MMP1, NR1H3, PLTP, and SCD had a lower overall survival rate. In addition, to validate our previous findings, we used the immunohistochemical data in the HPA database to explore the expression of these model molecules in BRCA tissues and normal breast tissues. Since some model molecules have not yet been included in the HPA database, we show the immunohistochemistry results of CPT1A, NR1H3, CYP27A1, SCD, SORBS1, and PLTP. The results showed that the expression of CPT1A, SCD, and PLTP molecules in BRCA tissues was significantly higher than that in normal breast tissues, and the expressions of NR1H3, CYP27A1, and SORBS1 molecules in BRCA tissues were significantly lower than those in normal breast tissues (Figures 8(a)–8(f)). These results prove that our previous findings are correct.

## 4. Discussion

Globally, cancer is the leading cause of human death [45, 46]. Previous studies have shown that cancer cells are characterized by their ability to increase indefinitely and grow out of control, evade the body's immune surveillance, perform energy metabolism in the form of glycolytic metabolism, exhibit dedifferentiation, and grow in a dominant clonal manner for invasion and migration [47]. It is precisely because these biological characteristics of cancer cells have been separated from the essential characteristics and evolutionary processes inherent in the body's normal cells. Researchers speculate that there must be decisive factors and a genetic basis. The earliest molecular understanding of tumors came from David Paul von Hansemann and Theodor Boveri [48]. They found that cancer cell division is accompanied by abnormal chromosomes, speculating that the formation of cancer and abnormal cell clones is caused by abnormal genetic material. With the in-depth exploration of cancer by modern researchers, it has been shown that diseases such as cancer are genetic diseases, and the occurrence and development of cancer are not the results of one or several uncontrolled cancer/tumor suppressor genes. Its development often involves biological pathways and multiple gene clusters with specific functions. Therefore, current scientific research is increasingly observing cancer progress at the level of biological pathways [49–51].

In response to the urgency and complexity of cancer research, TCGA project came into being. The primary research purpose of this project is to discover the information and technical tools needed to decode the molecular structure of cancer cells and enhance human understanding of the genetic basis of cancer. Through genome analysis technology, including the application of large-scale gene sequencing technology, we are fully committed to researching the molecular genetic basis of cancer. The ultimate goal is to improve the ability of humans to diagnose, treat, and prevent tumors. In this study, with the help of the precious data in TCGA database, we have the opportunity to explore above the level of a biological pathway. Based on TCGA database, this study focused on cancer-related PPAR signaling pathways. We used various biological information analysis methods to explore and successfully establish a prognostic-related risk model using PPAR signaling pathway-related genes in BRCA. This risk model comprises ten genes APOA2, APOA5, APOC3, CPT1A, CYP27A1, MMP1, NR1H3, PLTP, SCD, and SORBS1. These risk model genes all play essential roles in the malignant progression of BRCA. Among them, APOA5 has the most significant coefficient in the risk model, indicating that APOA5 is a critical prognostic factor in BRCA and has an important reference value for judging the prognosis of patients. In particular, we also used the immunohistochemical data in the HPA database to verify our previous findings. The verification results also show that our previous findings are correct.

MMP1, as a member of the matrix metalloproteinase family (MMPs), is an enzyme involved in extracellular matrix remodeling and plays an essential regulatory role in the interaction between tumor cells and the tumor microenvironment [52]. Previously, Hu et al. reported that BMP-6 plays a role in inhibiting BRCA metastasis by regulating the secretion of MMP1 in the tumor microenvironment [53]. Juncker-Jensen et al. said that MMP1 stimulates PAR1 to promote vascular growth and primary tumor metastasis [54]. The study by Langenskiöld et al. showed that MMP1 is an independent prognostic factor affecting the survival rate of patients with colorectal cancer [55]. In a study of East Asian breast patients, researchers found that lower expressions of APOA5 and APOC3 were associated with higher ESTIMATE immune scores, which means a large number of tumor-infiltrating immune cells [56]. CPT1A is a protein-coding gene, and its encoded protein is the key enzyme of the carnitine-dependent transport across the mitochondrial inner membrane. Its overexpression will increase the rate of fatty acid *β*-oxidation, thereby providing the cell with more ATP to maintain the various physiological activities. In breast cancer, a previous study has shown that miR-328-3p regulates tumor cell stemness by affecting the CPT1A-FAO axis in BRCA cells, thereby playing a pivotal role in BRCA metastasis [57]. In addition, inhibiting the expression of CPT1A in prostate cancer, nasopharyngeal carcinoma, breast cancer, or lung adenocarcinoma cells can increase the sensitivity of cancer cells to hormone-blocking chemotherapy or radiotherapy [58–62].

NR1H3, also known as LXR*α*, plays an essential role in cholesterol and lipid homeostasis. It can also interact with the ubiquitin E3-ligase protein complex containing the BRCA1-associated RING domain 1 (BARD1) effect [63]. In breast cancer, NR1H3 was identified as an antiproliferative and adipogenic factor in breast cancer cells and proved that the antiproliferative effect of NR1H3 is independent of lipid biosynthesis [64, 65]. CYP27A1 is mainly involved in cholesterol metabolism, fat synthesis and metabolism, steroid synthesis and metabolism, sterol metabolism, and other biological processes. It converts cholesterol into 27-hydroxycholesterol, maintains cholesterol homeostasis in the body, and catalyzes the biological activation of vitamin D3. The biologically active form of VD3 can induce cancer cell apoptosis and inhibit tumor growth [66, 67]. The results of previous studies indicate that the abnormal expression of CYP27A1 is closely related to the prognosis of a variety of tumors [68–70]. Alfaqih et al. found that the low expression of the CYP27A1 gene in prostate cancer is associated with survival rate and high tumor stage. Silencing the expression of the CYP27A1 gene can slow down the growth rate of prostate cancer cells in vitro and transplanted tumors [68]. Liang et al. showed that by downregulating the expression of CYP27A1 in bladder cancer and further achieving rapid proliferation of bladder cancer cells [71]. In addition, in a study of premenopausal estrogen receptor-positive primary BRCA patients, researchers found that the abnormal expression of CYP27A1 can be used as a prognostic indicator for such BRCA patients [72]. In breast cancer, Song et al. found that knocking down the expression of SORBS1 promotes the EMT process and reduces the sensitivity to chemicals, especially cisplatin, by inhibiting p53 in BRCA cells [73]. In addition, another study showed that miR-142-5p could regulate BRCA progression by targeting SORBS1 [74]. Up to now, APOA2, SCD, and PLTP molecules have not been deeply explored in BRCA. Therefore, in subsequent scientific research, we should explore the potential mechanisms of APOA2, SCD, and PLTP molecules in BRCA. In addition, in future clinical diagnosis and treatment, we can use the PPAR signaling pathway-related risk model to calculate the risk value of each patient. For BRCA patients in the high-risk group, we should give more intensive treatment, more detailed examinations, and more frequent reexaminations to prolong the survival time of BRCA patients in the high-risk group as much as possible.

## 5. Conclusion

In short, our study may still have some obvious shortcomings, such as the lack of single-center or multicenter clinical data verification. Therefore, we look forward to more large-sample, multicenter clinical studies to support this study in the future. However, based on TCGA database, we have established a prognostic-related risk model for BRCA patients using genes related to the PPAR signaling pathway and established a nomogram that can predict the overall survival of BRCA patients in five, seven, and ten years. In particular, for this risk model, we conducted a series of pancancer analyses and verified it with GEO and HPA databases. Therefore, this research can provide valuable data for future studies of the PPAR signaling pathway in BRCA.

## Data Availability

The data used to support the findings of this study are available from the corresponding author upon request.

## Abbreviations

TCGA: The Cancer Genome Atlas
BRCA: Breast cancer
CNV: Copy number variation
SNV: Single-nucleotide variant
OS: Overall survival
GSCA: Gene Set Cancer Analysis
GDSC: Genomics of Drug Sensitivity in Cancer
HPA: Human Protein Atlas
FABP1: Fatty acid binding protein 1
APOA5: Apolipoprotein A5
APOC3: Apolipoprotein C3
CPT1A: Carnitine palmitoyltransferase 1A
MMP1: Matrix metallopeptidase 1
NR1H3: Nuclear receptor subfamily 1 group H member 3
CYP27A1: Cytochrome P450 family 27 subfamily a member 1
APOA2: Apolipoprotein A2
SCD: Stearoyl-CoA desaturase
SORBS1: Sorbin and SH3 domain containing 1
PLTP: Phospholipid transfer protein.
